# The influence of obesity and gender on outcome after reversed L-shaped osteotomy for hallux valgus

**DOI:** 10.1186/s12891-019-2823-6

**Published:** 2019-10-15

**Authors:** Stephan H. Wirth, Niklas Renner, Richard Niehaus, Jan Farei-Campagna, Marcel Deggeller, Fabrice Scheurer, Katie Palmer, Thorsten Jentzsch

**Affiliations:** 1 Department of Orthopaedics, Balgrist University Hospital, University of Zurich, Forchstrasse 340, 8008 Zurich, Switzerland; 2 University of Zurich, Zurich, Switzerland; 3 San Camillo Hospital IRCCS, Venice, Italy

**Keywords:** Hallux valgus deformity (HV), Reversed L-shaped osteotomy (ReveL), Long plantar arm osteotomy, Body mass index (BMI), Gender, Radiological relapse, Improvable patient satisfaction

## Abstract

**Background:**

Hallux valgus deformity (HV) affects around every fourth individual, and surgical treatment is performed in every thousandth person. There is an ongoing quest for the best surgical management and reduction of undesirable outcomes. The aim was to explore associations of obesity and gender with radiological and clinical outcome after reversed L-shaped osteotomy (ReveL) for HV.

**Materials and methods:**

This study was carried out in a retrospective cohort design at a single University Hospital in Switzerland between January 2004 and December 2013. It included adult patients treated with ReveL for HV. The primary exposure was body mass index (BMI) at the time of ReveL. The secondary exposure was gender. The primary outcome was radiological relapse of HV (HV angle [HVA] > 15 degrees [°]) at the last follow-up. Secondary outcomes were improvable patient satisfaction, complication, redo surgery, and optional hardware removal. Logistic regression analysis adjusted for confounders.

**Results:**

The median weight, height, and BMI were 66.0 (interquartile range [IQR] 57.0–76.0) kilograms (kg), 1.65 (IQR 1.60–1.71) metres (m), and 24.0 (IQR 21.3–27.8) kg/m^2^. Logistic regression analysis did not show associations of relapse with BMI, independent of age, gender, additional technique, and preoperative HVA (adjusted odds ratio [OR_adjusted_] = 1.10 [95% (%) confidence interval (CI) = 0.70–1.45], *p* = 0.675). Relapse was 91% more likely in males (OR_adjusted_ = 1.91 [95% CI = 1.19–3.06], *p* = 0.007). Improvable satisfaction was 79% more likely in males (OR_adjusted_ = 1.79 [CI = 1.04–3.06], *p* = 0.035). Hardware removal was 47% less likely in males (OR_adjusted_ = 0.53 [95% CI 0.30–0.94], *p* = 0.029).

**Conclusions:**

In this study, obesity was not associated with unsatisfactory outcomes after ReveL for HV. This challenges the previous recommendation that preoperative weight loss may be necessary for a successful surgical treatment outcome. Males may be informed about potentially higher associations with unfavourable outcomes. Due to the risk of selection bias and lack of causality, findings may need to be confirmed with clinical trials.

## Background

Hallux valgus deformity (HV) is defined by a lateral (valgus) deviation of the great toe (hallux) leading to a potentially painful medial bony prominence of the metatarsal joint [1]. It is found in about every fourth individual [2]. Pathophysiological mechanisms of HV are gender-specific, whereby women are more commonly affected [3, 4]. Older age and the use of constrictive shoe wear are also considered risk factors for HV [5–8]. Surgical treatment of HV is one of the most commonly performed procedures in orthopedics [9]. The prevalence of forefoot surgeries, most of which are for HV, is 0.8 per 1000 individuals [10]. After HV surgery, patients are more commonly less satisfied than after other common orthopaedic procedures (e.g. total knee arthroplasty) [11]. Consequently, there is an ongoing quest for the best surgical management and causes of undesirable outcomes.

Various surgical techniques for HV have been described [12]. In the distal metatarsal, (potentially biplanar) reversed L-shaped osteotomy (ReveL), the bone cut is performed in a short dorsal upright and long plantar level fashion, thereby, offering very good stable correction [13, 14].

On one hand, HV has been reported to be inversely associated with obesity, according to a population-based foot study of 3077 older adults, where Dufour et al. reported that a body mass index (BMI) > 30 kg per meters^2^ (kg/m^2^) was associated with a decrease in HV by 33% in males and 45% in females [15]. On the other hand, higher revision rates for complications after HV surgery have been associated with obesity in a retrospective comparative study of 49 obese and 403 non-obese patients, where Chen et al. reported a complication rate of 14% for the obese compared to 2% for the non-obese [16]. Furthermore, increased bodyweight is associated with higher plantar loading pressure patterns as previously reported by Butterworth et al. [17]. Thus, theoretically, more weight could potentially put more strain on a deformity or osteotomy site, leading to higher failure rates. Therefore, an association of obesity with the outcome after HV surgery needs further investigation, particularly in context with specific surgical techniques.

So far obesity and gender have not been studied in relation to unsatisfactory radiological and clinical outcomes after long plantar arm osteotomies. The aim of the current study is to explore the associations of obesity and gender with radiological and clinical outcome after reversed L-shaped osteotomy (ReveL) for HV with particular focus on the association of weight, height, and body mass index (BMI) with radiological relapse, improvable patient satisfaction, complication, surgical redo surgery, and hardware removal after ReveL. It was hypothesized that obesity and male gender would be associated with worse radiological and clinical outcomes.

## Materials and methods

### Study design

This retrospective cohort study was done as a part of a Master’s thesis and further results about other study aims are reported in other publications [18, 19].

### Setting

At the Balgrist University Hospital in Switzerland, between January 2004 and December 2013, patient charts were studied.

### Ethics

The approval of an ethics committee was acquired by the local ethics committee (KEK ethics reference: 2015–0480), permitting this study with many patients without requiring individually signed informed consent.

### Surgical technique

Details of the surgical technique including an illustrative figure [20] have been described elsewhere [18, 19]. In short, surgical indication consisted of pain due to HV, ReveL was performed using a short dorsal vertical bone cut (starting around a centimeter [cm] proximal to the joint between the metatarsus and phalanx on the dorsum of the foot) before transitioning into a long horizontal bone cut at the bottom of the foot (eventually reaching the bone on the bottom around 4 cm proximal to the joint), and postoperative rehabilitation included ambulation as tolerated in a so-called postoperative shoe with a stiff sole for around 1 month [21].

### Participants

Patients, who underwent primary ReveL for HV and were ≥ 18 years were included. Our database (KISIM; CISTEC AG, Zurich, Switzerland) was examined for the term “hallux valgus” yielding 1977 cases. Exclusion criteria were refusal to participate in any research (*n* = 9), missing documentation (*n* = 43), other surgery (*n* = 789), primary revision (*n* = 69), no use of ReveL for HV (*n* = 218), and a particular reason for the selected screw number (*n* = 22). Finally, 827 cases were considered in this study.

### Variables

BMI (continuous data) was the primary exposure variable. It was calculated with the following formula: weight (kilogram [kg]) / height (metres [m])^2^. The secondary exposure variable was gender (male or female). As shown in the tables, other recognised exposure variables and/or potential confounders/effect modifiers were taken into account.

Radiological relapse of HV, defined as HV angle [HVA] > 15 degrees (°), at last radiological follow-up was the primary outcome variable [22]. Secondary clinical outcome variables consisted of improvable patient satisfaction, complication, redo surgery, and optional hardware removal [18, 19]. After thorough chart review, improvable patient satisfaction was considered as dissatisfaction and improvement (in comparison to being satisfied and very satisfied) to simplify analysis. In our clinic, self-reported satisfaction is assessed in follow up consultations and our retrospective patient chart review retrieved these data. Complication was noted to be present if any of the following were observed during follow-up: infection, necrosis, pseudarthrosis, Morbus Sudeck, and revision. Redo surgery was defined as any surgical procedure that addressed complications of ReveL. Optional hardware removal was considered if there this was performed upon a patient’s wish instead of an absolute indication for surgery.

### Data sources and measurements

The patient charts were reviewed on a local database (KISIM) and imaging software (IMPAX 6.4.0.6010 and IMPAX Orthopaedic Tools, Agfa-Gevaert N.V. [Agfa], Mortsel, Belgium). Data were anonymously entered into REDCap (version 6.11.5; Vanderbilt University, Nashville, TN, United States of America [USA]) before cleaning was performed with Microsoft Excel (version 2010; Microsoft Corporation, Redmond, WA, USA) and Stata/IC (version 13.1; StataCorp LP, College Station, TX, USA).

### Study size

In order to detect a clinically relevant difference in radiological relapse of 10% at *p* ≤ 0.05 and a power of 80%, ≥199 cases per group were needed.

### Statistical methods

Data are presented as medians (interquartile range [IQR]). For easier analysis, continuous data were changed into binary values. Two different categories were chosen for BMI to investigate a potential linear trend. Body mass index was changed into a binary variable (< 30.0 and ≥ 30.0 kg/m^2^) as well as categorical variable (< 25.0, 25.0–29.9, 30.0–34.9, or ≥ 35.0 kg/m^2^). For more clarity, analysis was also performed for a lower cut-off for the BMI (25 instead of 30 kg/m^2^).

Categorical variables were assessed with the chi-squared test (and score test for trend of odds) and the odds ratio (OR) (95% [%] confidence interval [CI]) are presented. Continuous variables were assessed with the Wilcoxon rank sum test. At the beginning, age and preoperative HVA were defined as confounders since they often affect the prevalence of diseases, including HV [3–6, 23]. Other confounders were considered if crude and adjusted estimates varied by at least 10%. Adjusted Mantel-Haenszel estimates were computed. A logistic regression with Wald tests was utilized for a causal model. Statistical significance was set at *p* < 0.05. Stata/IC was used for the analysis (StataCorp LP).

## Results

### Participants

Seventeen (2.1%) cases were lost during follow-up and 810 (97.9%) cases analysed, at a median follow-up of 12.0 (IQR 4.0–19.0) months. There were missing data for weight (*n* = 9 [1.1%]), height (*n* = 9 [1.1%]), and BMI (*n* = 11 [1.4%]). This was due to missing information in patient charts.

### Exposure variables

The median weight, height, and BMI were 66.0 (IQR 57.0–76.0) kg, 1.65 (IQR 1.60–1.71) m, and 24.0 (IQR 21.3–27.8) kg/m^2^ (Table 1). The majority were female (*n* = 717 [88.5%]).

**Table 1:** Exposure variables of cases with hallux valgus deformity (HV) treated with reversed L-shaped osteotomy (ReveL) (*n* = 810)

Variable	Category	N_total_ (%)	N_category_ (%)	Median (IQR)
Weight (kg)		801 (98.9)^a^		66.0 (57.0–76.0)
Height (m)		801 (98.9)^a^		1.65 (1.60–1.71)
BMI (kg/m^2^)		799 (98.6)^a^		24.0 (21.3–27.8)
	< 25.0		453 (56.7)	
	≥25.0		346 (43.3)	
	< 30.0		679 (85.0)	
	≥30.0		120 (15.0)	
Gender		810 (100.0)		
	Female		717 (88.5)	
	Male		93 (11.5)	

*Abbreviations*: *N* Number, % Percent, *IQR* Interquartile range, *kg* kilograms, *m* metres, *BMI* Body mass index

^a^Missing data for weight, height, and body mass index due to missing information in patient chart

### Multivariate analysis of primary outcome

According to the logistic regression model, there was no association between radiological relapse of HV after ReveL and BMI (OR_adjusted_ = 1.10 [CI = 0.70–1.45], *p* = 0.675) (Table 2). These findings remained when the BMI cut-off was changed from 30 to 25 kg/m^2^. However, logistic modelling revealed strong evidence that radiological relapse of HV was 91% more likely in males than females (OR_adjusted_ = 1.91 [CI = 1.19–3.06], *p* = 0.007).

**Table 2:** Logistic regression model for particular factors associated with radiological relapse of hallux valgus deformity (HV) (HV angle [HVA] > 15°) and BMI as well as gender after reversed L-shaped osteotomy (ReveL) for HV (*n* = 799)

Main effect of variable	Stratum-specific effect of variable	Category	Adjusted OR (95% CI)^a^	*P*-value^b^
BMI (kg/m^2^)				
		< 30.0	1.00 (reference)	
		≥30.0	1.10 (0.70–1.75)	0.675‡
Gender				
		Female	1.00 (reference)	
		Male	1.91 (1.19–3.06)	0.007

*Abbreviations*: *OR* Odds ratio, % percent, *CI* Confidence interval, *BMI* Body mass index, *kg* kilograms, *m* metres

^a^Adjusted for confounders and effect modifiers: age, gender, preoperative hallux valgus angle, number of screws, additional surgical technique for hallux valgus, time period, and body mass index

^b^Wald test

^‡^The *p*-value remained insignificant if the BMI category was set to < 25.0 versus ≥25.0 (adjusted OR = 1.04 [95% CI 0.74–1.48], *p* = 0.817)

### Multivariate analysis of secondary outcomes

No evidence of an association of improvable patient satisfaction with BMI (OR_adjusted_ = 1.24 [95% CI 0.74–2.08], *p* = 0.420) was recorded. Improvable satisfaction was 79% more likely in males (OR_adjusted_ = 1.79 [CI = 1.04–3.06], *p* = 0.035).

There was no evidence for associations of complications with BMI (OR_adjusted_ = 0.77 [95% CI 0.29–2.06], *p* = 0.603) and gender (OR_adjusted_ = 2.05 [95% CI 0.89–4.71], *p* = 0.090).

No evidence for associations of redo surgery with BMI (OR_adjusted_ = 1.12 [95% CI 0.35–3.61], *p* = 0.843) and gender (OR_adjusted_ = 2.23 [95% CI 0.67–7.46], *p* = 0.192) was observed.

No evidence of an association of optional hardware removal with BMI (OR_adjusted_ = 0.87 [95% CI 0.54–1.38], *p* = 0.541) was seen. There was strong evidence that optional hardware removal was 47% less likely in males than in females (OR_adjusted_ = 0.53 [95% CI 0.30–0.94], *p* = 0.029).

Furthermore, obese patients had a higher preoperative intermetatarsal angle (12.7° [BMI < 30 kg/m^2^] vs 13.8° [BMI ≥ 30 kg/m^2^], *p* < 0.001).

## Discussion

According to the findings of this single-centre cohort study, there is no independent association of BMI with radiological relapse of HV, improvable patient satisfaction, complication, redo surgery, or optional hardware removal. However, there is evidence that being male is independently associated with increased radiological relapse of HV after ReveL for HV. Being male is also independently associated with an increase in having improvable patient satisfaction as well as less optional hardware removals. Furthermore, as a novel finding, this study shows that obese patients are more likely to have larger intermetatarsal angles.

There is sparse literature about the surgical technique presented herein using a long plantar arm osteotomy for HV and study sizes were rather small [21, 24–28]. There is, to the best of knowledge, only one other study about the association of obesity and outcome after surgery for HV (*n* = 452) [16]. A distal chevron osteotomy was used for milder deformities and a scarf osteotomy was utilized for more severe deformities; if needed, a Lapidus and/or Akin osteotomy were added. Higher redo surgery rates for obese patients were reported. The conclusions were that obese (*n* = 49) patients with a BMI of at least 30.0 kg/m^2^ had an increased risk of redo surgery than controls (*n* = 403), but that obese patients should still receive HV surgeries since they had comparable functional outcome scores after 2 years. Our results did not find any differences between obese and non-obese patients; neither for redo surgeries, nor for patient satisfaction. This may be attributed to different surgical techniques. In ReveL osteotomies, there is high intrinsic mechanical stability, which may overpower the forces of increased weight in obese patients. A study by Vienne et al. compared the load to failure of three different surgical techniques (Scarf, modified Chevron, and ReveL osteotomies) [29]. They found that the ReveL osteotomy combined the advantages of both other osteotomies in that it displayed high corrective power (contact area of 163 [±20mm^2^]) and stability (relative load to failure [compared to non-osteotomized sawbones] 87%). However, as reported by Nery et al., HV deformities in males are commonly hereditary (positive family history in 68% versus 36% in females; bilateral in 71%) and occur at an earlier onset with higher severity than in women [30]. This genetic contribution may have a larger influence on the course of disease than the surgical technique. These findings are important because the male subgroup is usually underreported in the literature as the female-to-male ratio is as high as 17:1 [31].

No evidence of an association between BMI with radiological relapse of HV may be present since deforming forces that act on the first ray may be lower than those needed for failure of fixation. In other words, the additional weight in obese patients may not be strong enough to deform an osteotomy site. Similarly, no evidence of an association between BMI with clinical outcome may have been found since obesity may not be associated with less soft tissue irritation, swelling, and foreign body sensation. Therefore, obesity may not be associated with unsatisfactory outcomes after ReveL for HV.

Gender-specific differences in the pathophysiology of HV are known and resilient intrinsic factors counteracting surgical correction of HV could possibly be the reason for increased rates of radiological relapse of HV and improvable patient satisfaction in males [30, 31]. Although not validated by our results, it could be speculated that males may potentially be less cautious in the postoperative rehabilitation period increasing their risk for unfavourable outcomes. Males may have lower rates of hardware removal due to the fact that they may be more likely to accept imperfections, such as palpable screws.

Although a limited number of surgeons in Switzerland use ReveL for HV, HV is very common, which renders these results important to this population. Since only patients from a single-centre in Switzerland were included, these findings may be limited to this population and not inevitably generalised for other populations. A novel aspect is that this study considers potential confounders of the association between BMI and radiological as well as clinical outcome variables for independent associations of exposures with outcomes in a large patient cohort. To overcome risks of a type II errors and underestimation of differences, another well-conducted non-inferiority trial could assess if obese patients actually have similar outcomes.

### Limitations

There were different follow-up times for cases, which may have introduced surveillance bias. Since follow-up is very good in Switzerland, as seen is this study, the last follow-up may be equivalent to the final result. Furthermore, patient satisfaction was assessed according to patient charts retrospectively. Also usually documented well, future studies may use standardized score sheets such as the Foot and Ankle Outcome Score (FAOS) [32].

Although the hypotheses were known to the investigators, outcome assessment should not have been largely influenced since the association with BMI had never been studied before and the direction of the outcome was completely unknown. Detailed training in data collection and specific measurement techniques, group-practicing, and discussions limited measurement errors and differential misclassification.

Estimates may have changed if other risk factors, such as different surgeons had been kept constant. Other potential risk factors, such as genetic susceptibility, were not taken into account, and may have confounded or interacted with crude estimates. Although a large sample size was chosen, some confidence intervals were very wide suggesting a lack of power in some subgroups. Moreover, there is always a role of chance and type I errors that cannot be completely eliminated. Finally, despite all of the effort described in this limitations section, potential risks for confounding, bias, and random error remain due to the retrospective cohort design of this study.

## Conclusions

In this study, obesity was not associated with unsatisfactory outcomes after ReveL for HV. This challenges the previous recommendation that preoperative weight loss may necessary for a successful surgical treatment outcome. Males may be informed about potentially higher associations with unfavourable outcomes. Due to the risk of selection bias and lack of causality, findings may need to be confirmed with clinical trials.

## Data Availability

All data analyzed during this study are included in this article.

## Abbreviations

%: Percent
°: Degrees
BMI: Body mass index
CI: Confidence interval
cm: Centimeters
HV: Hallux valgus deformity
HVA: Hallux valgus angle
IQR: Interquartile range
kg: Kilogram
m: Meter
mm: Millimeters
MTP: Metatarsophalangeal
OR: Odds ratio
ReveL: Reversed L-shaped osteotomy
